# Lack of a peroxiredoxin suppresses the lethality of cells devoid of electron donors by channelling electrons to oxidized ribonucleotide reductase

**DOI:** 10.1371/journal.pgen.1006858

**Published:** 2017-06-22

**Authors:** Susanna Boronat, Alba Domènech, Mercè Carmona, Sarela García-Santamarina, M. Carmen Bañó, José Ayté, Elena Hidalgo

**Affiliations:** 1 Oxidative Stress and Cell Cycle Group, Universitat Pompeu Fabra, Barcelona, Spain; 2 Departamento de Bioquímica y Biología Molecular, Universitat de València, Valencia, Spain; MRC Laboratory of Molecular Biology, UNITED KINGDOM

## Abstract

The thioredoxin and glutaredoxin pathways are responsible of recycling several enzymes which undergo intramolecular disulfide bond formation as part of their catalytic cycles such as the peroxide scavengers peroxiredoxins or the enzyme ribonucleotide reductase (RNR). RNR, the rate-limiting enzyme of deoxyribonucleotide synthesis, is an essential enzyme relying on these electron flow cascades for recycling. RNR is tightly regulated in a cell cycle-dependent manner at different levels, but little is known about the participation of electron donors in such regulation. Here, we show that cytosolic thioredoxins Trx1 and Trx3 are the primary electron donors for RNR in fission yeast. Unexpectedly, *trx1* transcript and Trx1 protein levels are up-regulated in a G1-to-S phase-dependent manner, indicating that the supply of electron donors is also cell cycle-regulated. Indeed, genetic depletion of thioredoxins triggers a DNA replication checkpoint ruled by Rad3 and Cds1, with the final goal of up-regulating transcription of S phase genes and constitutive RNR synthesis. Regarding the thioredoxin and glutaredoxin cascades, one combination of gene deletions is synthetic lethal in fission yeast: cells lacking both thioredoxin reductase and cytosolic dithiol glutaredoxin. We have isolated a suppressor of this lethal phenotype: a mutation at the Tpx1-coding gene, leading to a frame shift and a loss-of-function of Tpx1, the main client of electron donors. We propose that in a mutant strain compromised in reducing equivalents, the absence of an abundant and competitive substrate such as the peroxiredoxin Tpx1 has been selected as a lethality suppressor to favor RNR function at the expense of the non-essential peroxide scavenging function, to allow DNA synthesis and cell growth.

## Author summary

The essential enzyme ribonucleotide reductase (RNR), the rate-limiting enzyme of deoxyribonucleotide synthesis, relies on the thioredoxin and glutaredoxin electron flow cascades for recycling. RNR is tightly regulated in a cell cycle-dependent manner at different levels. Here, we show that cytosolic thioredoxin Trx1 is the primary electron donor for RNR in fission yeast, and that *trx1* transcript and protein levels are up-regulated at G1-to-S phase transition. Genetic depletion of thioredoxins triggers the DNA replication checkpoint up-regulating RNR synthesis. Furthermore, deletion of the genes coding for thioredoxin reductase and dithiol glutaredoxin is synthetic lethal, and we show that a loss-of-function mutation at the peroxiredoxin Tpx1-coding gene acts as a genetic suppressor. We propose that in a mutant strain compromised in reducing equivalents, the absence of an abundant and competitive substrate of redoxins, the peroxiredoxin Tpx1, has been selected as a lethality suppressor to favor channeling of electrons to the essential RNR.

## Introduction

Cysteine residues are not very abundant in proteins, but they are over-represented in functional regions of proteins, such as surfaces and catalytic centers [1]. The thiol group of cysteines is subject of post-translational modifications altering its redox state; several of these oxidation states are reversible, such as sulfenic acid and disulfides. In particular, reversible thiol to disulfide switches happen as a consequence of cellular responses to oxidative stress, and several proteins with reactive cysteine residues undergo oxidations as part of their catalytic cycles (for a review, see [2]). Cells are provided with two major systems meant to control the thiol-disulfide status, the thioredoxin (Trx) and the glutaredoxin/glutathione (Grx/GSH) systems.

Trxs and Grxs catalyze thiol-disulfide exchange reactions, and share a motif known as the Trx fold [3]. Thermodynamically, both types of reductants use as the ultimate electron donor NADPH [4]. Electrons are therefore transferred from NADPH to final substrates through gradients in redox potentials. In the case of Trxs, Trx reductase is the intermediate between NADPH and Trx, while GSH reduces oxidized Grxs, GSH reductase being the link between NADPH and oxidized GSH.

Trx was first identified in 1964 as an electron donor for *Escherichia coli* ribonucleotide reductase (RNR), an enzyme required for DNA synthesis [5]. Grx was later discovered as an alternative electron donor for the same enzyme in *E*. *coli* mutants lacking Trx [6]. Many reports indicate that there is cross-talk between both branches of these electron transfer systems and certain redundancy, but it is also clear that there is substrate specificity.

From then onwards, it became clear that these oxido-reductases regulate a wide number of processes in eukaryotic and prokaryotic organisms, apart from DNA synthesis and repair, including antioxidant defense and redox regulation, sulfur metabolism or apoptosis; the substrates of Trxs and Grxs mediating these effects are peroxiredoxins (Prxs), GSH peroxidases, methionine sulfoxide reductases, phosphoadenylyl sulfate (PAPS) reductase or RNRs (for reviews on these and other functions of the electron donor cascades, see [2,7–12]). In most cell types, the only substrate of electron donors which is essential for survival (and not only for specific cellular processes such as cysteine biosynthesis or oxidative stress tolerance) is RNR.

RNR catalyzes the reduction of ribonucleosides into deoxyribonucleosides, and is therefore essential to provide the building blocks, deoxyribonucleotides (dNTPs), during DNA replication and repair. In eukaryotes, class Ia RNRs are composed of a large subunit, α, containing the catalytic site and two allosteric effector binding sites, that control which substrate is reduced (specificity site) as well as the rate of reduction (activity site) [13,14], and a small subunit, β, containing a stable diferric-tyrosyl radical cofactor (oxygen is required for the assembly of the diferric-tyrosyl radical cofactor in RNRs), which initiates nucleotide reduction through the transient oxidation of a cysteine to a thiyl radical in the catalytic site of the α subunit. During this process, two local cysteines in the large subunit provide reducing equivalents, and the disulfide bond generated between them, after isomerizing within the same α monomer towards a solvent-exposed position, is reduced by Trx or Grx to yield active RNR.

Balanced and sufficient pools of dNTPs have to be present during S phase of the cell cycle, and also to assist in DNA repair. In fact, several studies suggest that a correct supply of dNTPs during DNA replication is important for genome stability and for the prevention of cancer [15,16]. Inhibition of RNR activity by the radical scavenger hydroxyurea (HU) and other compounds has been used as a chemotherapeutic strategy of numerous cancer types [16,17]. RNRs are tightly regulated through many different mechanisms, which include allosteric and oligomeric regulation, transcription of the α and/or β-coding genes to modulate protein levels, inhibition of RNR catalytic activity and regulation of the subcellular localization of the RNR subunits (for reviews on RNR regulation, see [18,19]). In *Schizosaccharomyces pombe*, Cdc22 and Suc22 are the large and small subunits of RNR, respectively [20]. Most studies concerning regulation of fission yeast RNR activity have centered on the RNR inhibitor Spd1, which affects activity and subunit localization of α and β [21–23], and on the up-regulation of *cdc22* transcription during the S phase and after DNA damage (for a review, see [19]). While the *suc22* transcript does not fluctuate with the cell cycle, transcription of *cdc22* is up-regulated by the MBF transcription factor, which triggers expression of genes required for the S phase [20,24,25]. Regarding regulation of *cdc22* expression by checkpoint kinases under stress conditions, treatment with HU, which inhibits RNR, decreases the available pool of dNTPs and causes the formation of stalled replication forks and the activation of the DNA replication checkpoint driven by the Rad3 and Cds1 kinases; activated Cds1 phosphorylates and inactivates the Yox1 transcriptional repressor, promoting MBF-dependent *cdc22* expression [26]. Regarding the regulation of RNR by cofactors and post-transcriptional modifications, changes in RNR subunit localization in response to iron bioavailability have been recently demonstrated in budding yeast [27].

Nevertheless, very little is known about the redox-dependent cell cycle regulation of RNR activity [28]. In fact, the identity of the *S*. *pombe* electron transfer components required for RNR recycling is unknown. Here, we identify Trx1 and Trx3 as the main electron donors of fission yeast RNR, we demonstrate that Trx1 expression is actually up-regulated at S phase at the transcript and protein levels, and that in the absence of Trx1 and Trx3 the DNA replication checkpoint is activated. With the expectation that a complete block of electrons flow should drive to cell lethality by blocking RNR at its oxidized form unless a continuous synthesis of RNR is triggered, we have managed to generate a synthetic lethal combination by deleting the Trx reductase and the Grx1-coding genes. A spontaneous suppressor of this synthetically lethal phenotype is a frame-shift mutation at the beginning of *tpx1*, the gene coding for the most abundant consumer of electrons in the cell, the Prx Tpx1. Our experiments suggest that in the triple knockout strain *Δtrr1 Δgrx1 Δtpx1*, the elimination of the main sink of electron favors the reduction of the essential substrate RNR.

## Results

### Trxs are the preferred electron donors of *S*. *pombe* RNR large subunit Cdc22

**Fig 1:**
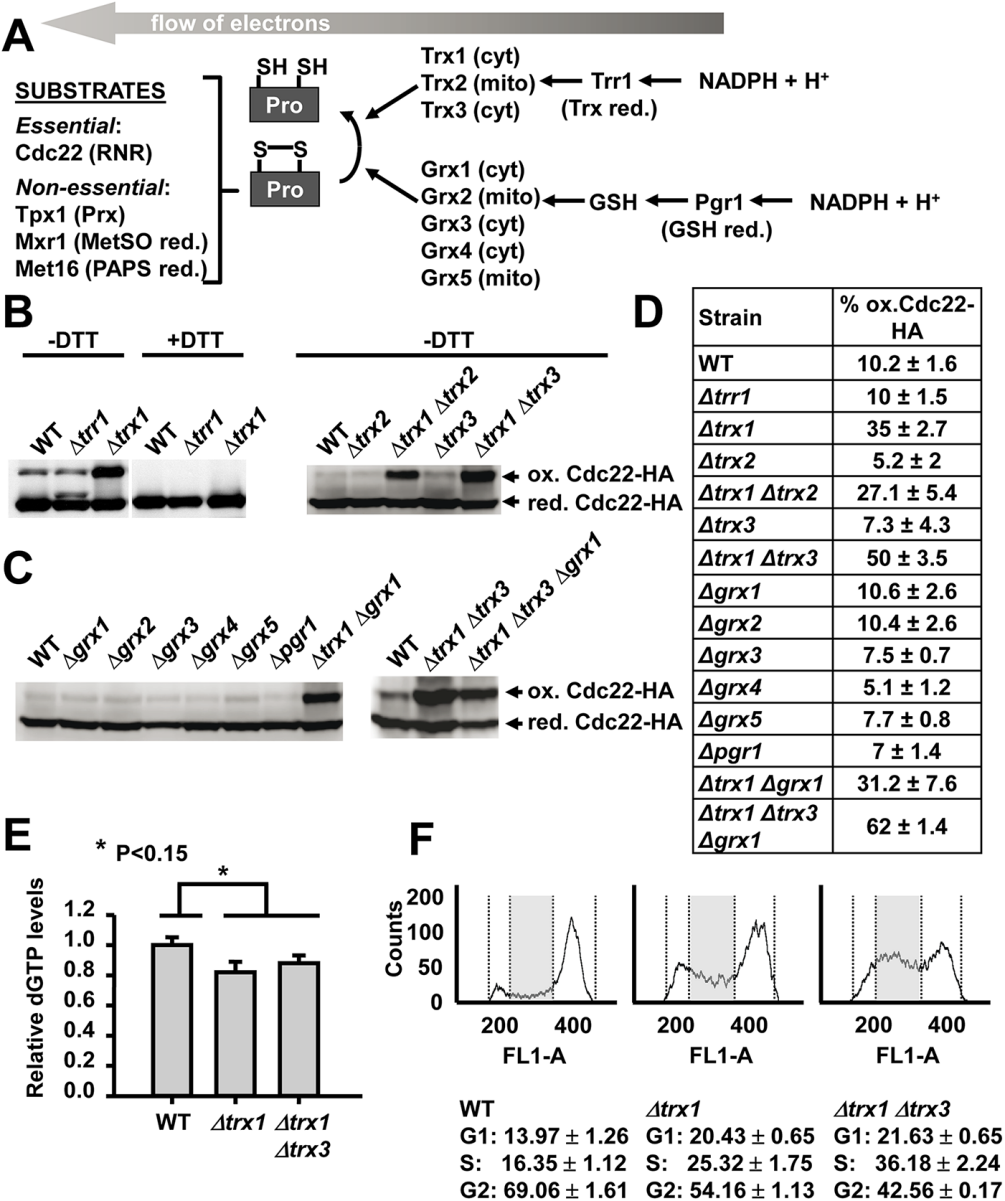
The large subunit of RNR, Cdc22, is substrate of cytosolic Trxs. (A) Schematic representation of the electron flow cascades in fission yeast. Trxs 1 to 3, and Grxs 1 to 5 are indicated, with their proposed subcellular localization (cytosolic, cyt; mitochondrial, mito). Trx reductase (Trr1) and GSH reductase (Pgr1) are also indicated, as well as some substrates of these electron donors cascades, such as the large subunit of RNR Cdc22, the main Prx Tpx1, methionine sulfoxide reductase (MetSO red.) Mxr1 or phosphoadenylyl sulfate reductase (PAPS red.) Met16. (B) Trxs 1 and 3 are involved in the recycling of Cdc22. TCA extracts from YE cultures of strains SB104 (WT), SB105 (*Δtrr1*), SB106 (*Δtrx1*), SB134 (*Δtrx2*), SB133 (*Δtrx1 Δtrx2*), SB115 (*Δtrx3*) and SB121 (*Δtrx1 Δtrx3*), all of them carrying an *HA*-tagged version of *cdc22* at their endogenous loci, were obtained and processed by SDS-PAGE in the presence or absence of DTT and analyzed by Western blot using antibody against HA. Reduced/active (red. Cdc22-HA) and oxidized/inactive Cdc22 (ox. Cdc22-HA) are indicated. (C) The Grx system is not required to reduce Cdc22. TCA extracts from strains SB104 (WT), SB140 (*Δgrx1*), SB131 (*Δgrx2*), AD178 (*Δgrx3*), SB111 (*Δgrx4*), AD181 (*Δgrx5*), AD104 (*Δpgr1*), SB141 (*Δtrx1 Δgrx1)*, SB121 (*Δtrx1 Δtrx3)* and SB308 (*Δtrx1 Δtrx3 Δgrx1)*, all of them carrying HA-tagged version of Cdc22, were processed by non-reducing SDS-PAGE as described in B. (D) Percentage of oxidized Cdc22-HA in the indicated strains. Quantification was performed as described in Materials and Methods. SEM from three independent experiments are shown. (E) dGTP levels in wild-type and Trx mutants. Strains SB117 (WT), SB137 (*Δtrx1*) and SB138 (*Δtrx1 Δtrx3*) were grown in YE and dGTP levels were determined by the DNA polymerase-based enzymatic assay. The values are represented relative to those of the wild-type strain. Error bars (SEM) from three independent experiments are shown. (F) DNA content analysis of isolated nuclei. Samples taken from asynchronous cultures of strains 972 (WT), MJ15 (*Δtrx1*) and SG248 (*Δtrx1 Δtrx3*) were ethanol fixed. Nuclei were isolated and DNA content was determined as described in Materials and Methods. Histograms represent the number of cells (Counts) with different FL1-A (DNA content), 200 corresponding to 1C and 400 corresponding to 2C. A representative histogram of each strain is shown. Cells in S phase are indicated as a gray rectangle in each of the histograms. Gates for G1, S and G2/M are indicated with dashed lines. The percentage of cells in G1, S and G2 has been calculated from three independent experiments and is shown below each histogram. SEM from three independent experiments are shown.

*S*. *pombe* contains three genes coding for Trxs [29] and one for Trx reductase, *trr1* (Fig 1A). Trx1 is the main cytoplasmic Trx [30]; Trx2 is localized to the mitochondria [31]; and Trx3/Txl1 has cytoplasm localization, although it is also associated with the proteasome [32–34]. Trx1 and, to a minor extent, Trx3 are the electron donors of the Prx Tpx1, essential for aerobic scavenging of peroxides and for signal transduction [35–37]. Thus, while cells lacking Trx1 are extremely sensitive to hydrogen peroxide (H_2_O_2_), Trx2 and Trx3 appear to be dispensable for the defense against oxidative stress [35]. Regarding the other branch of the disulfide reductases, fission yeast expresses only two dithiol Grxs, cytosolic Grx1 and mitochondrial Grx2, several monothiol Grxs such as Grx4 (involved in the iron starvation response) [38–40], endoplasmic reticulum-located Grx3 [41] and mitochondrial Grx5 (involved in mitochondrial iron-sulfur cluster assembly) [42], and one GSH reductase, Pgr1 (Fig 1A). Pgr1 has been reported to be essential at least during aerobic growth [43], but cells lacking the reductase can grow under semi-anaerobic conditions. These two cascades have to recycle enzymes which suffer disulfide formation as part of their catalytic functions, such as the essential Cdc22 and the non-essential Tpx1, Mxr1 or Met16 (Fig 1A).

To test the role of these cascades in the turnover of the large RNR subunit, Cdc22, we combined single or multiple deletion mutations with the expression of a tagged version of the protein, Cdc22-HA. This modification, performed at the chromosomal locus, did not affect cell fitness or cell tolerance to the RNR inhibitor HU (S1 Fig). As shown in Fig 1B, a DTT sensitive, slow migrating band was detected using anti-HA immune-blotting of extracts from asynchronous cultures of wild-type cells expressing Cdc22-HA, corresponding to 10.2 ± 1.6% of total Cdc22; the sensitivity to the dithiol DTT indicates that the slower migrating band corresponds to a disulfide-containing RNR. In extracts from cells lacking Trx reductase, a band between oxidized and reduced Cdc22 was detected, probably a transient intermediate between RNR and its electron donor. Importantly, cells lacking cytosolic Trx1, but not the mitochondrial Trx2, displayed 35 ± 2.7% of Cdc22 in its oxidized form. The lack of Trx3 did not significantly affect the amount of Cdc22 disulfide form, but it enhanced the ratio of oxidized-to-reduced form (50 ± 3.5%) in a *Δtrx1* background. On the contrary, disruption of the Grx branch by deletion of the genes coding for Grx1-to-Grx5, or GSH reductase, Pgr1, did not exacerbate disulfide accumulation, unless added to the *Δtrx1 Δtrx3* background (Fig 1C). The percentages of Cdc22 oxidation in these and other mutants of the Trx and Grx branches are indicated in Fig 1D.

To confirm that Trx deficiencies have an effect on Cdc22 activity, we measured dNTP levels of asynchronous wild-type, *Δtrx1* and *Δtrx1 Δtrx3* cultures (Fig 1E for dGTP, and S2A Fig for dATP), and detected small but significant decreases in the absence of Trxs. We also measured the percentage of cells at G1, S and G2 phases in asynchronous cultures from these cells. As shown in Fig 1F, cells lacking Trx1 and, to a larger extent, Trx1 and Trx3 displayed an enlarged population of cells at S phase, indicating that these strains completed DNA replication slower than wild-type cells.

### Transient disulfide accumulation of Cdc22 during S phase is reversed by Trx1 and Trx3

**Fig 2:**
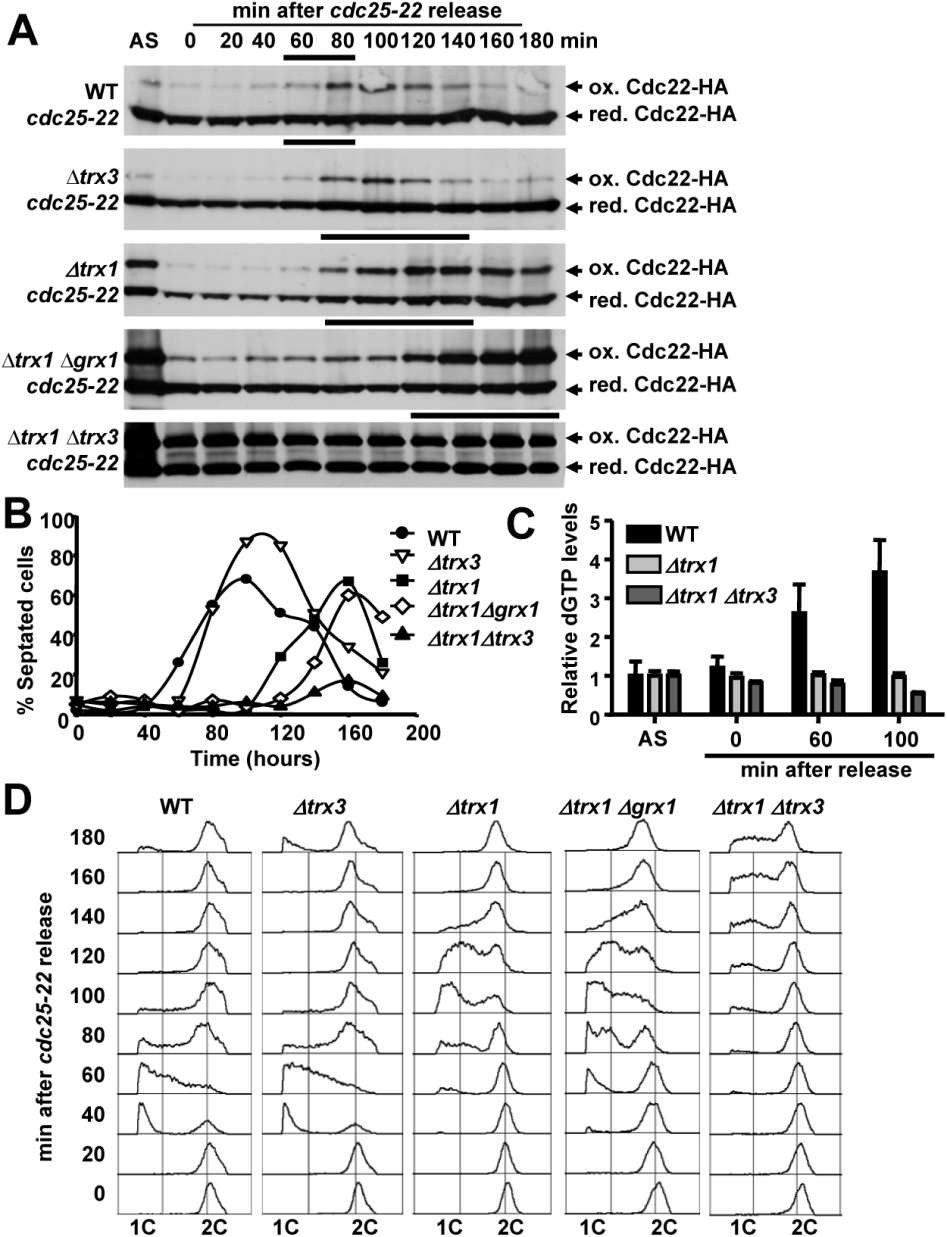
Oxidation of Cdc22 occurs during S phase in synchronous cultures and its cycling is dependent on Trx1 and Trx3. (A) Analysis of TCA protein extracts from YE cultures synchronized using the *cdc25-22* mutation. Strains SB117 (WT), SB142 *(Δtrx3)*, SB137 *(Δtrx1)*, SB141 *(Δtrx1 Δgrx1)* and SB138 *(Δtrx1 Δtrx3)*, carrying the temperature sensitive *cdc25-22* allele and expressing Cdc22-HA, were arrested in late G2 by a temperature shift to 36°C for 4 h, and then synchronously released from the arrest at 25°C. TCA protein extracts of asynchronous (AS) cell cultures, from arrested cells (time 0) and after release from the block (20 to 180 min) were analyzed as in Fig 1B. The solid black line at the top of each panel indicates the time points where the majority of the cells were in S phase, according to D. (B) Septation index was measured from samples taken at the indicated time points. (C) dGTP levels of cell cultures as in A at the indicated times points were determined and represented as in Fig 1E. (D) DNA content analysis of isolated nuclei. Samples taken from synchronized cultures of A were ethanol fixed, nuclei were isolated and DNA content analyzed by flow cytometry as described in Fig 1F.

We synchronized cultures of cells expressing Cdc22-HA and carrying mutations in several components of the reducing cascades by means of the *cdc25-22* allele. Cdc25 is the G2-to-M activating phosphatase of the cyclin-dependent kinase of *S*. *pombe*, Cdc2. Upon shift to the non-permissive temperature, *cdc25-22* cells are arrested at the G2/M transition and after dropping the temperature, cells are synchronically released from the arrest. As shown in Fig 2A, Cdc22-HA oxidation cycles in wild-type cells and in cells lacking Trx3, with a transient peak from 60 to 120 minutes after the release. This peak overlaps with that of the septation index (Fig 2B), which in fission yeast is concomitant with S phase. The peaks of Cdc22-HA oxidation and septation index are delayed and more sustained in cells lacking Trx1 (or Trx1 and Grx1). Strikingly, in cells devoid of cytosolic Trxs, *Δtrx1 Δtrx3*, Cdc22-HA oxidation does not cycle and the protein is maintained at its oxidized form at high levels, around 50–60%. In fact, these cells do not have a clear septation peak.

Next, and to confirm that the activity of Cdc22 was compromised in the mutants lacking Trxs, we measured dNTP levels in the previous synchronous cultures. As shown in Fig 2C (dGTP) and S2B Fig (dATP), while the levels of dNTPs increased in wild-type cells during cell cycle progression (60 and 100 min after release), cells lacking Trx1 or Trx1 and Trx3 displayed a reduction of dNTPs levels, pointing that these strains could have compromised DNA synthesis. In fact, when we analyzed the DNA content from the synchronous cultures, we indeed observed a delayed and extended S phase in *Δtrx1* cells (Fig 2D). This is even more noticeable in cells in which all the cytosolic Trxs were absent: in *Δtrx1 Δtrx3* S phase was not detected by FACS until 120 min after the release, which represents 60 minutes of delay when compared to wild-type cells.

### The *trx1* gene is up-regulated at the G1-to-S transition of the cell cycle

**Fig 3:**
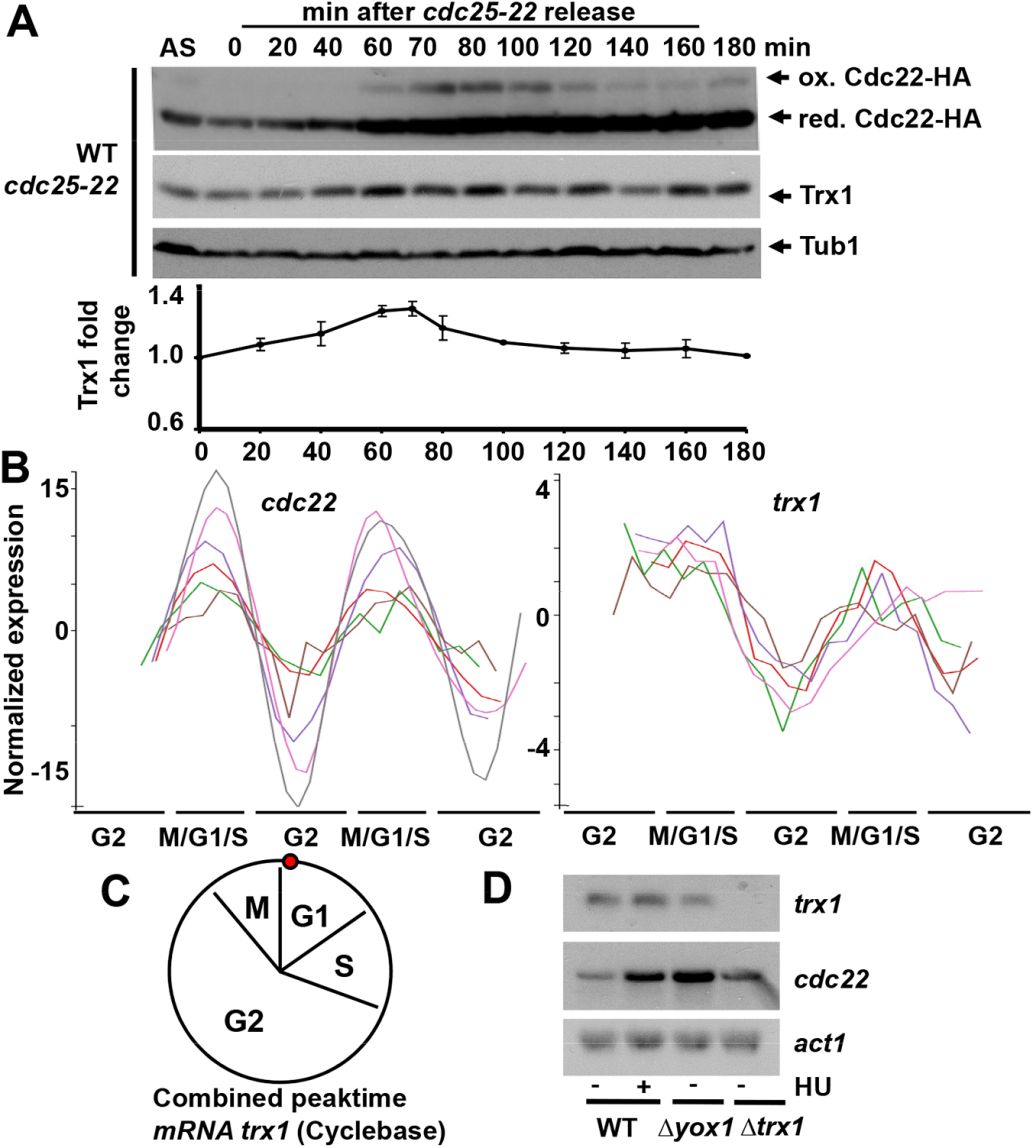
The Trx1 protein and *trx1* mRNA levels are up-regulated during G1/S. (A) Strain SB117 (WT *cdc25-22 cdc22-HA*) was processed as in Fig 2A and analyzed with antibodies against HA or Trx1, as indicated. Tubulin was used as a loading control. The graph below shows the Trx1 fold change relative to the arrested cells (0 min). Error bars (SEM) from three independent experiments are shown. (B) Normalized expression of *cdc22* and *trx1* mRNA through the cell cycle according to the database Cyclebase 3.0 (http://cyclebase.org/). The expression values of *cdc22* and *trx1* through the cell cycle from six independent experiments were first transformed to log2, then the mean value was substracted over all time points, and finally, the expression values were all normalized. (C) Combined peaktime of the *trx1* transcript in a regular fission yeast cell cycle. Data obtained from the database Cyclebase 2.0. (D) Up-regulation of *trx1* is not dependent on the MBF complex. Total RNA prepared from untreated (-) or HU-treated (+) (180 min, 10 mM HU) cultures of strains 972 (WT), JA804 *(Δyox1)* and MJ15 *(Δtrx1)* was analyzed by Northern blot with probes for *trx1* and *cdc22*, a MBF-dependent gene; *act1* was used as a loading control.

Once confirmed that Cdc22 oxidation is exacerbated during catalysis, we tested whether the expression of its main electron donor was also up-regulated during S phase. As shown in Fig 3A, Trx1 protein levels were enhanced at S phase as determined in extracts from block and release experiments. To test whether this protein up-regulation was dependent on a transcriptional event, we used Cyclebase, a repository of published cell cycle experiments [44], to interrogate genome-wide studies on block and release experiments performed in fission yeast. As shown in Fig 3B, there is a small but consistent cell cycle regulation of *trx1 mRNA*. The combined peaktime of all published datasets occurs at the beginning of G1 (Fig 3C), a bit later than other S phase transcripts such as *cdc22*, *cdc18*, *yox1* or *nrm1* (S3 Fig). All these genes are up-regulated by the MBF transcription factor. To test whether the increase of *trx1 mRNA* is dependent on MBF, we analyzed its transcript levels upon HU treatment or in cells lacking the MBF repressor Yox1. As shown in Fig 3D, *trx1* transcription does not seem to depend on the MBF complex, contrary to *cdc22*. Future work will help us elucidating who triggers the accumulation of *trx1 mRNA* at G1-to-S transition.

### The absence of Trx1 and Trx3 triggers the DNA replication checkpoint and induces transcription of *cdc22*

**Fig 4:**
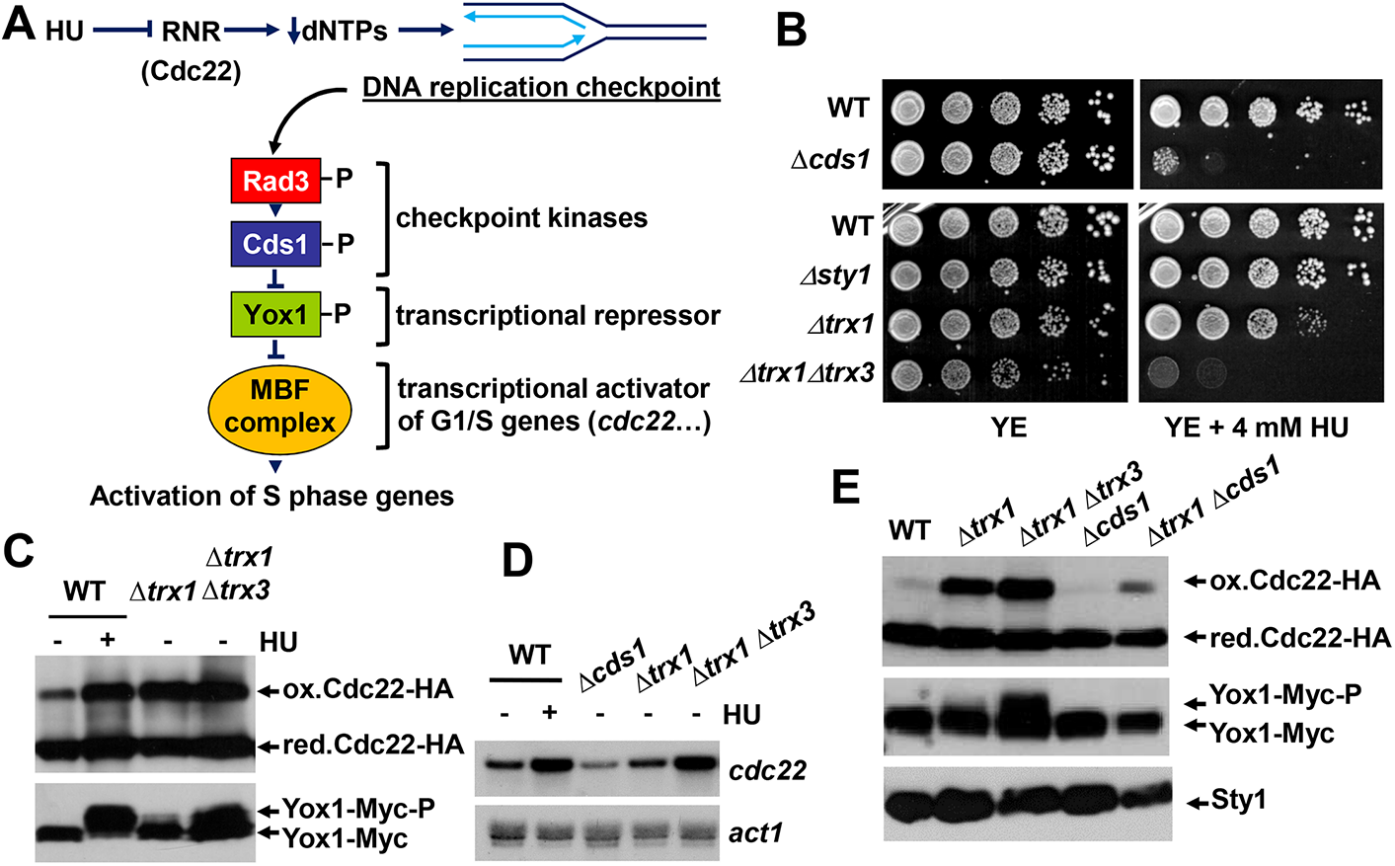
Trx deficiency triggers the DNA replication checkpoint. (A) Scheme showing the activation by HU, an RNR inhibitor, of the Rad3-Cds1 checkpoint cascade. The decrease in the total concentration of dNTPs induces stalling of the replication fork and the activation of the checkpoint kinases Rad3 and Cds1. One of the substrates of activated Cds1 is Yox1, a transcriptional repressor of the MBF complex, an activator of G1/S genes. Among other genes, transcription of *cdc22* is triggered to compensate the decline in dNTP concentrations. (B) Serial dilutions of YE cultures of strains 972 (WT), JA1158 *(Δcds1)*, AV18 *(Δsty1)*, MJ15 *(Δtrx1)* and IC76 *(Δtrx1 Δtrx3)* were spotted on YE agar plates without or with 4 mM HU, and grown for 3 days at 30°C. (C) TCA extracts prepared from untreated (-) or HU-treated (+) (180 min, 10 mM HU) YE cultures of strains SB149 (WT), SB150 *(Δtrx1)* and SB153 *(Δtrx1 Δtrx3)*, all expressing Cdc22-HA and Yox1-13Myc, were analyzed as in Fig 1B with antibodies against HA or Myc, as indicated. (D) Total RNA prepared from untreated (-) or HU-treated (+) (180 min, 10 mM HU) YE cultures of strains 972 (WT), JA943 *(Δcds1)*, MJ15 *(Δtrx1)* and SG248 *(Δtrx1 Δtrx3)* was analyzed as in Fig 3D with probes for *cdc22* and *act1*, used as a loading control. (E) TCA extracts from untreated YE cultures of SB149 (WT), SB150 *(Δtrx1)*, SB153 *(Δtrx1 Δtrx3)*, SB200 *(Δcds1)* and SB199 *(Δcds1 Δtrx1)* were analyzed as in Fig 1B. Sty1 levels were used as loading control.

Inhibition of RNR activity by HU treatment triggers a DNA replication stress, probably through the decrease in dNTP concentrations and stalling of DNA polymerase at replication forks. In *S*. *pombe*, the DNA replication checkpoint is driven by the Rad3 and Cds1 kinases. One target of this cascade is the transcriptional repressor Yox1, which after phosphorylation by Cds1 is released from the MBF complex and its S phase promoters [26,45] (Fig 4A).

To test whether RNR inhibition by Trx deficiency can trigger replication stress, we first tested whether cells lacking Trx1 and Trx3 are sensitive to the presence of the RNR inhibitor HU. As shown in Fig 4B, *Δtrx1* and *Δtrx1 Δtrx3* cells are moderately and severely sensitive to HU, respectively, highlighting their defects in DNA synthesis. In wild-type cells, HU treatment exacerbates the accumulation of total and oxidized Cdc22-HA, as well as of the inhibitory phosphorylation of Yox1 (Fig 4C). Both Trx mutant strains, but specially *Δtrx1 Δtrx3*, display constitutive activation of the Rad3-Cds1 checkpoint cascade, as demonstrated by the presence of phosphorylated Yox1 even in the absence of HU stress in this strain background (Fig 4C), and by the enhanced levels of *cdc22* mRNA under basal conditions (Fig 4D). We generated a *Δtrx1 Δcds1* strain to demonstrate that the weak phosphorylation of Yox1 in *Δtrx1* cells is dependent on Cds1 (Fig 4E). *Δtrx1 Δtrx3* deletions are synthetic lethal with deletion of *cds1*, while a triple *Δtrx1 Δtrx3 Δchk1* mutant is viable (S4 Fig); Chk1 is the effector kinase of the DNA damage checkpoint. We propose that the survival of cells lacking both Trxs depends at least partially on the Cds1-dependent transcriptional up-regulation of the *cdc22* gene (Fig 4A).

### *Δtrr1 Δgrx1*, inactivating both the Trx and the Grx electron donor branches, is synthetic lethal

**Fig 5:**
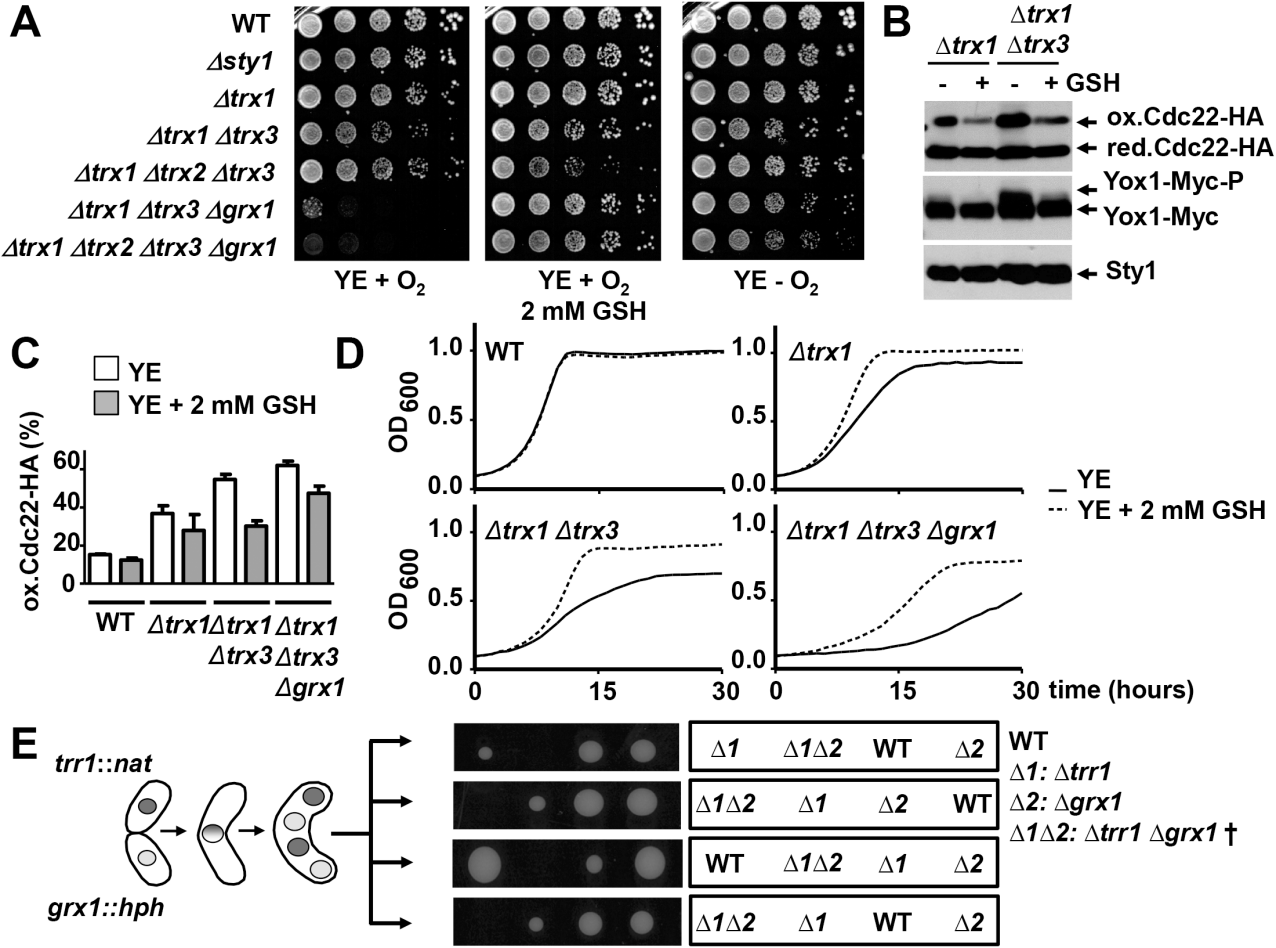
Deletion of *trr1* and *grx1* is synthetic lethal. (A) Survival of several multiple deletion mutants in the Trx and Grx cascades, and growth recovery with GSH or semi-anaerobic conditions. Serial dilutions of YE cultures of strains 972 (WT), AV18 *(Δsty1)*, SG70 *(Δtrx1)*, SG248 *(Δtrx1 Δtrx3)*, IC146 *(Δtrx1 Δtrx2 Δtrx3)*, SB160 *(Δtrx1 Δtrx3 Δgrx1)* and SB226 *(Δtrx1 Δtrx2 Δtrx3 Δgrx1)* were spotted on YE plates in the presence or not of 2 mM GSH, and plates were grown at 30°C under aerobic (+O_2_) or semi-anaerobic (-O_2_) conditions. (B) TCA extracts prepared from untreated (-) or 2 mM GSH-treated (+) YE cultures of strains SB150 *(Δtrx1)* and SB153 *(Δtrx1 Δtrx3)*, all expressing Cdc22-HA and Yox1-13Myc, were obtained and analyzed as in Fig 4C. Sty1 levels were used as loading control. (C) Percentage of oxidized Cdc22-HA in SB104 (WT), SB106 (*Δtrx1)*, SB121 (*Δtrx1 Δtrx3*) and SB308 (*Δtrx1 Δtrx3 Δgrx1*) all expressing Cdc22-HA grown in the presence or absence of 2 mM GSH. Error bars (SEM) from three independent experiments are shown. (D) GSH partially suppresses the growth defects of liquid cultures of cells lacking electron donors. Growth of strains as in C was monitored by recording OD_600_ for a period of 40 hours of cultures containing (dashed lines) or not (solid lines) 2 mM GSH. (E) Double deletion of the *trr1* and *grx1* genes is synthetic lethal. Schematic representation of tetrad dissection. Strains SG166 (*Δtrr1; trr1*::*nat*) and SB34 (*Δgrx1; grx1*::*hph*) were crossed and the spores resulting from four tetrads were separated using a Singer tetrad micromanipulator and germinated on YE plates under semi-anaerobic conditions. The markers of the deletions were determined by replica-plating on antibiotic-containing plates (nourseothricin or hygromycin), and the final genotypes of each colony are indicated in the panel. No growth was detected for the double deletion (*Δ1 Δ2*: *Δtrr1 Δgrx1* †), which was inferred after subtracting all the markers accumulated in the remaining spores of each tetrad.

So far, we have demonstrated that Trxs are the main electron donors of Cdc22, and that cells lacking Trx1 and Trx3 have important defects and activate the replication checkpoint. Taking into account that RNR is an essential protein, we attempted to induce lethality by combining a number of deletions of genes coding for components of the electron pathway cascades (see Fig 1A). Many of the mutants displayed severe growth defects, which could often be rescued by growing the cells in semi-anaerobiosis or in the presence of exogenous GSH (S1 Table, Fig 5A). Indeed, exogenous addition of GSH was sufficient to decrease the ratio of oxidized-to-reduced Cdc22 in mutants lacking Trxs (Fig 5B and 5C) and to alleviate some of their growth defects in liquid media (Fig 5D). This suggests that the Grx-GSH branch is a back-up mechanism of reduction of the essential RNR.

After exhaustive combination of gene deletions, only two crosses lead to lethality in fission yeast: double deletions of the *trr1* (coding for Trx reductase) and *gcs1* (codes for glutamate-cysteine ligase, the rate-limiting enzyme on the GSH biosynthetic pathway) genes, or the double knock-out *trr1* and *grx1* (coding for the only dithiol cytosolic Grx) (Fig 5E). We propose that in this *Δtrr1 Δgrx1* strain background RNR would remain oxidized.

### Lack of Tpx1 restores the growth of cells lacking Trr1 and Grx1

**Fig 6:**
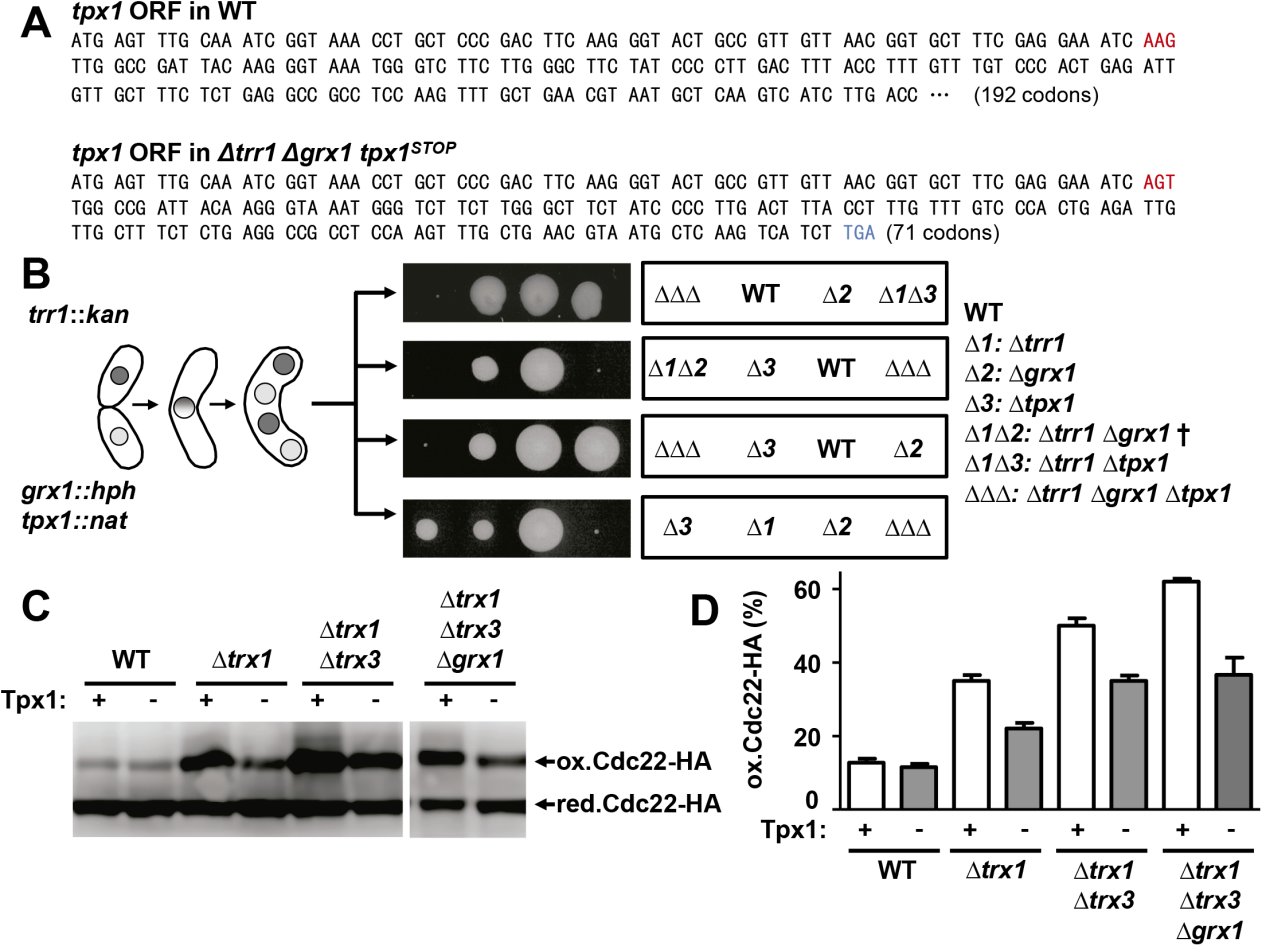
Loss-of-function of Tpx1 suppresses the lethal phenotype of a *Δtrr1 Δgrx1* double deletion. (A) Sequence comparison of the wild-type and mutant *tpx1* loci. The suppressor mutation is a nucleotide deletion at codon 26 (indicated in red in both loci) causing a frame shift leading to a truncated polypeptide of 70 amino acids (stop codon indicated in blue in the mutant allele). (B) Deletion of *tpx1* is a suppressor of the synthetic lethality of *Δtrr1 Δgrx1*. Strains SG169 (*trr1*::*kan)* and MC138 (*grx1*::*hph tpx1*::*nat*) were crossed and analyzed as in Fig 5E. Four very small colonies were isolated from four tetrads and recovered in GSH-containing plates grown under semi-anaerobic conditions. Again, no growth was detected for the double deletion (*Δ1 Δ2*: *Δtrr1 Δgrx1* †), which was inferred after subtracting all the markers accumulated in the remaining spores of the second tetrad. (C) TCA extracts from YE cultures of strains SB104 (WT), SB112 (*Δtpx1*), SB106 (*Δtrx1*), AD175 (*Δtrx1 Δtpx1*), SB121 (*Δtrx1 Δtrx3*), AD176 (*Δtrx1 Δtrx3 Δtpx1*), SB308 (*Δtrx1 Δtrx3 Δgrx1*) and AD172 (*Δtrx1 Δtrx3 Δgrx1 Δtpx1*), all of them carrying an *HA*-tagged version of *cdc22* at the endogenous locus, were analyzed as in Fig 1B. Reduced/active (red. Cdc22-HA) and oxidized/inactive Cdc22 (ox. Cdc22-HA) are indicated. (D) Graph shows the average percentage of oxidized Cdc22-HA from three independent experiments as in C. Error bars (SEM) are shown.

In spite of the results shown above, and using random spore selection, we unexpectedly obtained a single colony lacking Trr1 and Grx1 and therefore containing a suppressor mutation. To our surprise, we determined by sequence analysis that this mutation laid on the gene encoding the Prx Tpx1, introducing a one-base deletion at the 26^th^ codon of the open reading frame and subsequently a frame shift and a stop codon at position 71 (Fig 6A). To confirm that the suppressor mutation was linked to loss-of-function of Tpx1, we performed tetrad analysis to select a triple *Δtrr1 Δgrx1 Δtpx1* knock-out strain. As shown in Fig 6B by tetrad dissection, the double *Δtrr1 Δgrx1* is synthetic lethal, while tiny colonies of the *Δtrr1 Δgrx1 Δtpx1* strain were isolated under semi-anaerobic conditions and could be recovered on plates containing GSH. As shown in S5 Fig, the growth of this triple delete, *Δtrr1 Δgrx1 Δtpx1*, displays severe growth defects even under semi-anaerobic conditions, which can be partially overcome by GSH addition.

Tpx1 is probably the most demanding substrate of electron donors: there are more than 400,000 copies of the protein per cell [46], and Tpx1 is continuously catalyzing H_2_O_2_ detoxification during aerobic growth with the participation of Trx1, Trx3 and, probably, Grx1 [35,36]. The fact that strains such as *Δtrx1 Δtrx3 Δgrx1* grow better under semi-anaerobic condition (Fig 5A) is an indication that Tpx1 may be competing with Cdc22 for reducing equivalents in cells devoid of the main cytosolic electron donors: the levels of peroxides during semi-anaerobic metabolism are lower than in the presence of oxygen, and therefore Tpx1 is not cycling and demanding electrons to the same extent.

To demonstrate that the absence of Tpx1 could positively impinge on the reduced-to-oxidized ratio of RNR, we measured the amount of oxidized and reduced Cdc22-HA in different strains expressing or not Tpx1. As shown in Fig 6C and 6D, deletion of *tpx1* always reduced the percentage of oxidized Cdc22 in three different Trx-deficient strains. We conclude that *tpx1* deletion allows the channeling of electrons into the disulfide-bonded RNR, and this is particularly relevant in redoxin mutants.

**Fig 7:**
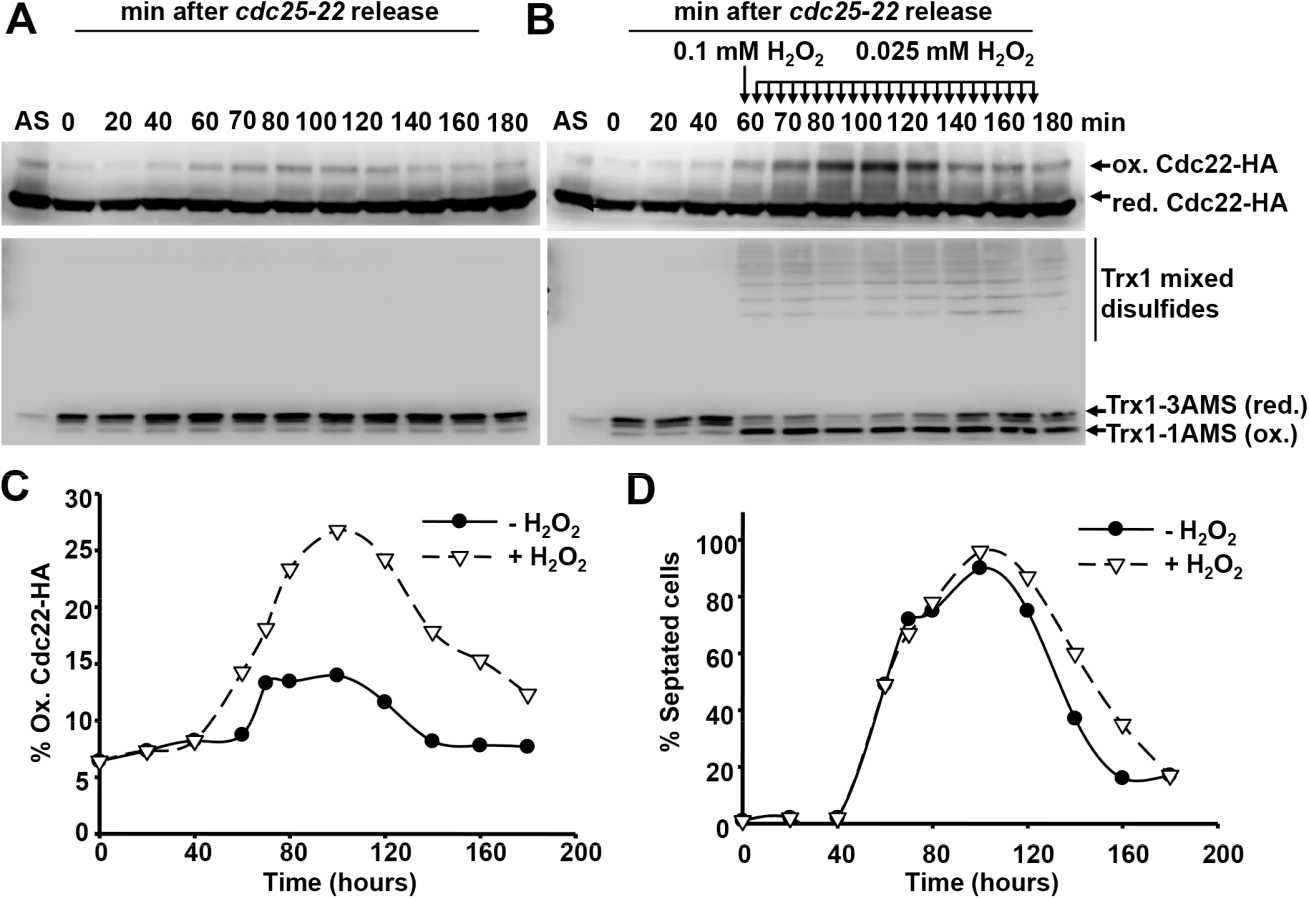
Transient depletion of reduced Trx1 in S phase by oxidized Tpx1 impairs timely recycling of Cdc22. (A, B) YE cultures of strain SB149 (WT *cdc25-22 cdc22-HA yox1-13myc*) were blocked and released as in Fig 2A. TCA extracts were prepared at the indicated timepoints and separated into two fractions. One fraction was used to visualize Cdc22-HA and was alkylated with iodoacetamide. The other fraction was used to visualize Trx1 and was alkylated with AMS. Alkylated extracts were analyzed by non-reducing gel electrophoresis and Western blotting with an HA antibody and with a specific Trx1 antibody, respectively. Reduced and oxidized Cdc22 are indicated. Reduced [Trx1-3AMS (red.)] and oxidized [Trx1-1AMS (ox.)] Trx1 are also indicated, as well as bands corresponding to transient mixed disulfides of Trx1 with its substrates, mainly Tpx1 (Trx1 mixed disulfides). (B) Same experiment performed as in A, but 0.1 mM H_2_O_2_ was added 60 minutes after release and then 0.025 mM H_2_O_2_ was added every 5 minutes thereafter. (C) Graph shows the percentage of oxidized Cdc22-HA at the indicated timepoints both in untreated (panel A) and H_2_O_2_ treated (panel B) cells. (D) Septation index from cells blocked and released in the presence (panel B) or absence (panel A) of H_2_O_2_.

To test whether the competition between Tpx1 and Cdc22 for reducing equivalents could occur in a wild-type background, we forced temporal depletion of reduced Trx1 by Tpx1 during S phase. Exhaustion of reduced Trx1 by Tpx1 can only be accomplished when the Prx is actively scavenging peroxides, but an excess of H_2_O_2_ triggers Tpx1 over-oxidation and avoids Trx1 depletion [35]. Therefore, we applied mild oxidative stress in a continuous manner to S phase cultures, by synchronizing wild-type cells expressing Cdc22-HA using the *cdc25-22* allele as shown in Fig 2, and adding or not 100 μM H_2_O_2_ at the onset of S phase, with subsequent additions of 25 μM every five minutes, to force Tpx1 oxidation and Trx1-dependent recycling (Fig 7). Trx1 oxidation was followed in extracts prepared in the presence of 4-acetamido-4′-maleimidylstilbene-2,2′-disulfonic acid (AMS) as described before [35]. AMS is a bulky thiol alkylating agent: while three moieties of AMS are incorporated in Trx1 when it is in the reduced form, only one AMS is incorporated when Trx1 is oxidized and two of its cysteine residues form a disulfide; slower migrating bands, corresponding to the transient mixed disulfides between Trx1 and its substrates, can also be detected by Western blot upon Trx1 oxidation. As shown in Fig 7A, the S phase-dependent oxidation of Cdc22 does not cause an apparent consumption of reducing equivalents, since the majority of Trx1 remains in the reduced form during the whole cycle. When a continuous addition of mild H_2_O_2_ is applied starting at 60 min (at the onset of S phase), a sustained oxidation of Trx1 is accomplished (Fig 7B), which is fully dependent on peroxide scavenging by Tpx1 [35]. Importantly, this Tpx1-dependent depletion of reduced Trx1 enhances the amount of oxidized Cdc22 (from 12% to 25%; Fig 7C), and the disulfide form is maintained for a longer period that in the absence of peroxides (Fig 7A, 7B and 7C). A small but significant cell cycle delay can be observed as a consequence of an elongated S phase, as demonstrated with the septation index (Fig 7D). In conclusion, if oxidative stress emerges during S phase, Tpx1 enzymatic activity jeopardizes the RNR-dependent synthesis of dNTPs through depletion of reduced Trx1.

## Discussion

Balanced pools of dNTPs have to be accumulated during S phase and after DNA damage and replication stress, and these DNA building blocks are synthesized on demand. In these two scenarios, replication and checkpoint activation, RNR activity is up-regulated through several different mechanisms. We have shown here that the catalytic disulfide formed at the large subunit of RNR, Cdc22, is reduced by the main cytosolic Trx, Trx1. Three important conclusions can be extracted from our work: first, the mRNA and protein levels of Trx1 are up-regulated during S phase, what demonstrates a new layer of regulation of RNR. Second, the other cytosolic Trx, Trx3, may support Trx1 in RNR recycling, so that cells lacking both electron donors suffer from severe replication stress which is partially overcome by the activation of the Rad3-Cds1 checkpoint. Third, the fitness phenotypes of mutants defective in electron donor capacity can be partially alleviated by depletion of another substrate of Trx1, the Prx Tpx1; elimination of an abundant competitor funnels electrons towards the essential RNR.

Regarding the cell cycle-dependent regulation of Trx1, all the experiments performed so far with synchronized *S*. *pombe* cultures highlight the smooth but consistent waves of *trx1* transcripts, with a G1 peaktime (http://cyclebase2.jensenlab.org/) (Fig 3B). We have discarded the participation of the main transcriptional activator of G1-S phase genes, the MBF complex, in *trx1* cycling (Fig 3D). Further work will be required to characterize this cell cycle-regulated event. Interestingly, it has recently been reported that colorectal cancer tissues display enhanced protein levels of both RNR and Trx1, and that inhibition of both proteins simultaneously produced a synergistic anti-proliferation effect in this model [47].

To the best of our knowledge, this is the first report demonstrating that eukaryotic cells carrying Trx deficiencies suffer from replication stress and constitutively trigger the DNA replication checkpoint. In *E*. *coli*, an interesting connection between electron donor supplies, activation of DNA replication by DnaA and transcription up-regulation of RNR was proposed by the group of Beckwith [48]. In *Saccharomyces cerevisiae*, it has been published that mutants lacking Trx1 and Trx2 display a longer S phase [49], that the total pool of dNTPs from asynchronous cultures is unaffected thanks to the novo synthesized RNR [50], but that dNTP levels of cells synchronized in S phase are significantly lower than those of wild-type cells [51]. In view of our results, this is probably due to a reduced pool of dNTPs to assist on DNA synthesis [28]. We show here that the checkpoint kinases Rad3 and Cds1 are constitutively active in *Δtrx1 Δtrx3* cells, and that this activation is required for the survival of this strain, since the triple *Δtrx1 Δtrx3 Δcds1* combination is lethal. We propose that in *Δtrx1 Δtrx3* cells the main source of active/reduced Cdc22 is *de novo* synthesized protein, which arises from the constitutive up-regulation of *cdc22* transcription in a Rad3-Cds1-Yox1 dependent manner.

The complete lack of Cdc22 recycling should drive cells to lethality. With this idea in mind, we have generated an extensive combination of deletion mutants in most components of the Trx and Grx branches, and one of these combinations resulted in synthetic lethality: *Δtrr1 Δgrx1*. The reason why other mutants, such as the quadruple *Δtrx1 Δtrx2 Δtrx3 Δgrx1* and *Δtrx1 Δtrx3 Δgrx1 Δgrx2* strains, could be isolated under semi-anaerobic conditions but not the aforementioned *Δtrr1 Δgrx1* strain is still intriguing to us. It can be speculated that the lack of Trx reductase is more pervasive that the elimination of its substrates due to the accumulation of oxidized Trxs, which may invert their role towards thiol oxidases [52,53], or which may bind to Trx substrates with the same affinity as reduced Trx [54] and block their reduction by other electron donors. A similar result has been reported in other organisms such as *E*. *coli*, where lethality or severe sickness can only be accomplished by deletion of the Trx reductase coding gene in combination with a defect in the Grx branch [55]. In *S*. *cerevisiae*, deletion of the *trx1*, *trx2*, *grx1* and *grx2* genes has been reported to be lethal [56].

Prxs are probably the most demanding cellular substrates of electron donors: they are continuously catalyzing peroxide scavenging during aerobic metabolism, and they are among the most abundant proteins in most cell types. We reported before that Trx1 and, secondarily, Trx3 are the main electron donors of Tpx1, and cells lacking both cytoplasmic Trxs (Trx1 and Trx3) display constitutively oxidized Tpx1 [35]. Cells lacking Tpx1 cannot grow aerobically on plates; however, strain *Δtrx1 Δtrx3* is still viable aerobically, suggesting a secondary role for the Grx/GSH system in Tpx1 reduction. Therefore, Tpx1 and Cdc22 compete for Trx1, Trx3 and, probably, another component(s) of the Grx/GSH cascade. This is not a problem in a wild-type background under most conditions: reduction of Tpx1 by Trx1 is hardly saturated, unless mild oxidative stress is applied, and only for a limited amount of time unless a continuous supply of peroxides is provided (Fig 7A and 7B); indeed, upon severe H_2_O_2_ stress, Tpx1 becomes hyper-oxidized to sulfinic acid and temporarily inactivated [35,37,57], which may be beneficial to avoid inhibition of RNR recycling. However, when electron donors become limiting by genetic interventions it is probably advantageous to promote reduction of an essential substrate, RNR, by eliminating a non essential one, a Prx. In fact, other processes improving the fitness of redoxin mutants as well as the oxidized-to-reduced ratio of RNR are semi-anaerobic growth (by minimizing the activity and electron consumption of Tpx1 in peroxide scavenging; Fig 5A) and GSH addition (by providing unlimited reducing power; Fig 5A and S5 Fig).

In our study, we present evidence for the existence of a novel electron donor for RNR, as the synthetic lethal phenotype of *Δtrr1 Δgrx1* mutant can be rescued by eliminating Tpx1, the major competitor substrate for electrons. It was similarly proposed by Grant and colleagues that PAPS reductase could have an alternative hydrogen donor to Trx1 and Trx2 in budding yeast, since a *Δtrx1 Δtrx2* strain grew on minimal media without sulphate under low-aeration growth conditions reducing the generation of reactive oxygen species [56], and probably minimizing the function of Prxs or GSH peroxidases. We have not identified yet the alternative electron donor of RNR in the *Δtrr1 Δgrx1 Δtpx1* background. There are still two or three genes in fission yeast coding for monothiol glutaredoxins (Grx3, Grx4, Grx5), which have been proposed to participate in processes other than disulfide reduction. At least in mammalian RNR, a GSH-mixed disulfide mechanism for Grx-mediated reduction of RNR has been described [58]. Whether *S*. *pombe* monothiol Grxs are mediating the channeling of electrons to RNR in a GSH-dependent manner, or whether GSH itself can reduce the disulfide in Cdc22 will have to be elucidated.

## Materials and methods

### Growth conditions, genetic manipulations and strains

Cells were grown in rich medium (YE) at 30°C as described previously [59]. When cells were crossed, we chose tetrad dissection or random spore analysis as indicated in the text. For tetrad analysis, asci were dissected by micromanipulation with a Singer Micromanipulator MSM 400 (Singer Instruments, UK). After growth of the dissected spores on YE agar plates under semi-anaerobic conditions, genetic markers were scored by replica-plating on YE-agar plates containing or not the indicated antibiotics, and placing the plates at 30°C under semi-anaerobic conditions in the presence or not of 2 mM GSH, as indicated. Anaerobic liquid cultures were grown in flasks filled to the top with medium at 30°C without shaking. Origins and genotypes of strains used in this study are outlined in Appendix S2 Table, and most of them were constructed by standard genetic methods. A strain with tagged Cdc22-HA, SB104, was constructed by replacing the *cdc22-YFP*::*kanMX6* cassette of strain AWS16 (*h+ cdc22-YFP*::*kanMX6 ade6-704 leu1-32 ura4-D18*, kindly provided by A. Carr), with a *cdc22-HA*::*natMX6* cassette and by cleaning the auxotrophies. The *natMX6* cassette in SB104 was replaced by *the hphMX6* cassette, resulting in strain SB110. Derived strains containing additional deletions were obtained by crossing SB104 or SB110 with the corresponding strains, and plating spores in appropriate media, with the exception of strain AD104 that was obtained by deletion of the *pgr1* gene in SB110. Strains with tagged Yox1-13Myc were obtained by crossing appropriate strains with JA778 *(h- yox1-13myc*::*kanMX6 ura4-D18)* or JA779 *(h+ yox1-13myc*::*kanMX6 ura4-D18)*.

### TCA extracts and immuno blot analysis

Modified trichloroacetic acid (TCA) extracts were prepared blocking thiols with either iodoacetamide or AMS and separated in non-reducing denaturing electrophoresis as previously described [37]. Only when indicated, the reducing agent dithiothreitol (DTT) was added to the sample buffer prior to electrophoresis. Cdc22-HA and Yox1-Myc were immuno-detected with monoclonal house-made anti-HA or anti-Myc antibodies, respectively. Trx1 was immuno-detected with anti-Trx1 polyclonal antibody [60]. Anti-Sty1 polyclonal antibody [36] was used as loading control. Relative quantification of protein levels in Western blots was performed by scanning membranes with a Licor 3600 CDigit Blot Scanner (Licor Inc., USA) and using the Image Studio 4.0 software.

### dGTP and dATP measurements

The dATP and dGTP levels were determined by the DNA polymerase-based enzymatic assay as described before [27]. In brief, the incorporation of dATP and dGTP into specific oligonucleotides, containing poly(AAAT) and poly(AAAC) sequences respectively, by the Klenow DNA polymerase was determined in the presence of excess [^3^H]-labeled dTTP.

### Flow cytometry analysis of DNA content in isolated nuclei

We followed a previously published protocol for determining DNA content on isolated nuclei [61]. Briefly, 1x10^7^ cells were fixed in 70% ethanol and nuclei were prepared. Isolated nuclei were treated with RNase A (37°C overnight) and DNA was stained in PBS solution containing 1 μM Sytox green.

### Cell cycle block and release experiments

Temperature-sensitive strains carrying the allele *cdc25-22* were cultured in YE at the permissive temperature (25°C) in a shaker water bath until reaching OD_600_ of 0.3, shifted to the non-permissive temperature (36°C) for 4 hours and then allowed to resume the cell cycle by growing them at 25°C during 3 hours as described [62]. Full arrest at G2/M was checked by microscopy. 5 ml aliquots were taken from non-arrested cells and at different times after release to prepare TCA extracts. Cell cycle progression was monitored with fluorescence microscopy by measuring the septation index of calcofluor-stained cells and by flow cytometry.

### RNA analysis

Total RNA from *S*. *pombe* YE cultures was obtained, processed and transferred to membranes as described previously [63]. Membranes were hybridized with the [α-^32^P]dCTP-labelled *cdc22*, *trx1* and *act1* probes, containing the complete open reading frames.

### HU and oxygen sensitivity assay on solid plates

For survival on solid plates, *S*. *pombe* strains were grown, diluted and spotted on YE plates containing or not HU at the indicated concentrations and plates were incubated at 30°C for 2–3 days as previously described [26]. To study the survival of strains on solid plates under aerobic or semi-anaerobic conditions, *S*. *pombe* strains were grown, diluted and spotted in YE, and plates were incubated at 30°C under aerobic or semi-anaerobic conditions. To grow cells in solid media in an semi-anaerobic environment, we incubated the plates at 30°C in a tightly sealed plastic bag containing a water-activated Anaerocult A sachet (Merck, Darmstadt, Germany) [36], or alternatively in a nitrogen-filled anaerobic chamber (Forma Anaerobic System, Thermo Electron Corp.). When indicated 2 mM GSH was added to YE agar plates.

### Growth curves

Yeast cells were grown in YE from an initial OD_600_ of 0.2, with or without the addition of 2 mM GSH, using an assay based on automatic measurements of optical densities, as previously described [64].

## Supporting information

S1 Fig C-terminal tagging of Cdc22 does not affect cell growth under basal or HU conditions. Serial dilutions of strains 972 (WT), JA1158 *(Δcds1)*, SB62 *(cdc22-YFP)*, SB104 *(cdc22-HA)* and SB212 *(cdc22-myc)* were spotted on agar plates without (YE) or with 2 and 4 mM HU, and grown for 3 days at 30°C.
(TIF)

S2 Fig Determination of dATP levels in wild-type and mutant strains. (A) dATP levels in wild-type and Trx mutants. Strains SB117 (WT), SB137 (*Δtrx1*) and SB138 (*Δtrx1 Δtrx3*) were grown in YE and dATP levels were determined by the DNA polymerase-based enzymatic assay. The values are represented relative to those of the wild-type strain. Error bars (SEM) from three independent experiments are shown. (B) dATP levels of cell cultures as in Fig 2A at the indicated times points were determined and represented as in Fig 1E.
(TIF)

S3 Fig The MBF-dependent *mRNAs* peak at the end of M phase or beginning of G1. Combined peaktime of the S phase transcripts *cdc22*, *yox1*, *cdc18*, *cdt1*, *cig2* and *nrm1*. Data obtained from the database Cyclebase 2.0.
(TIF)

S4 Fig Deletion of the Trx-coding genes does not interact genetically with the DNA damage checkpoint. (A) Scheme depicting the activation of Chk1 upon DNA damage. DNA damage activates the kinases Rad3 and Chk1. Cdc10, a member of the MBF complex, is then phosphorylated by Chk1, resulting in its release from chromatin and leading to repression of MBF dependent genes. (B) *Δchk1* does not display genetic interaction with *Δtrx1 Δtrx3*. Growth of 972 (WT), JA804 (*Δrad3*), SG248 (*Δtrx1 Δtrx3*) and AD151 (*Δtrx1 Δtrx3 Δchk1*) cells was monitored by recording the OD_600_ for a period of 30 hours. (C) Serial dilutions of strains 972 (WT), JA804 (*Δrad3*), JA795 (*Δyox1*), SG248 (*Δtrx1 Δtrx3*) and AD151 (*Δtrx1 Δtrx3 Δchk1*) were spotted on agar plates without (YE) or with 1 mM H_2_O_2_ or with 5 mM HU, and grown for 3 days at 30°C.
(TIF)

S5 Fig The triple delete *Δtrr1 Δgrx1 Δtpx1* displays severe growth defects. Serial dilutions of strains 972 (WT), SG4 (*Δtpx1*), SG248 (*Δtrx1 Δtrx3*), SB304 (*Δtrx1 Δtrx3 Δgrx1*) and MC144 (*Δtrr1 Δgrx1 Δtpx1*) were spotted on YE agar plates without or with 2 mM GSH under semi-anaerobic (-O_2_) or aerobic (+O_2_) conditions and grown for 3 days at 30°C.
(TIF)

S1 Table Phenotypes of strains mutated in different components of the Trx and Grx systems. (PDF)

S2 Table Strains used in this study. (PDF)
