# Supplementary figures and images for: Lack of a peroxiredoxin suppresses the lethality of cells devoid of electron donors by channelling electrons to oxidized ribonucleotide reductase
Source: PLoS Genet. 2017 Jun 22;13(6):e1006858. doi: 10.1371/journal.pgen.1006858 (PMC5501661; doi:10.1371/journal.pgen.1006858)

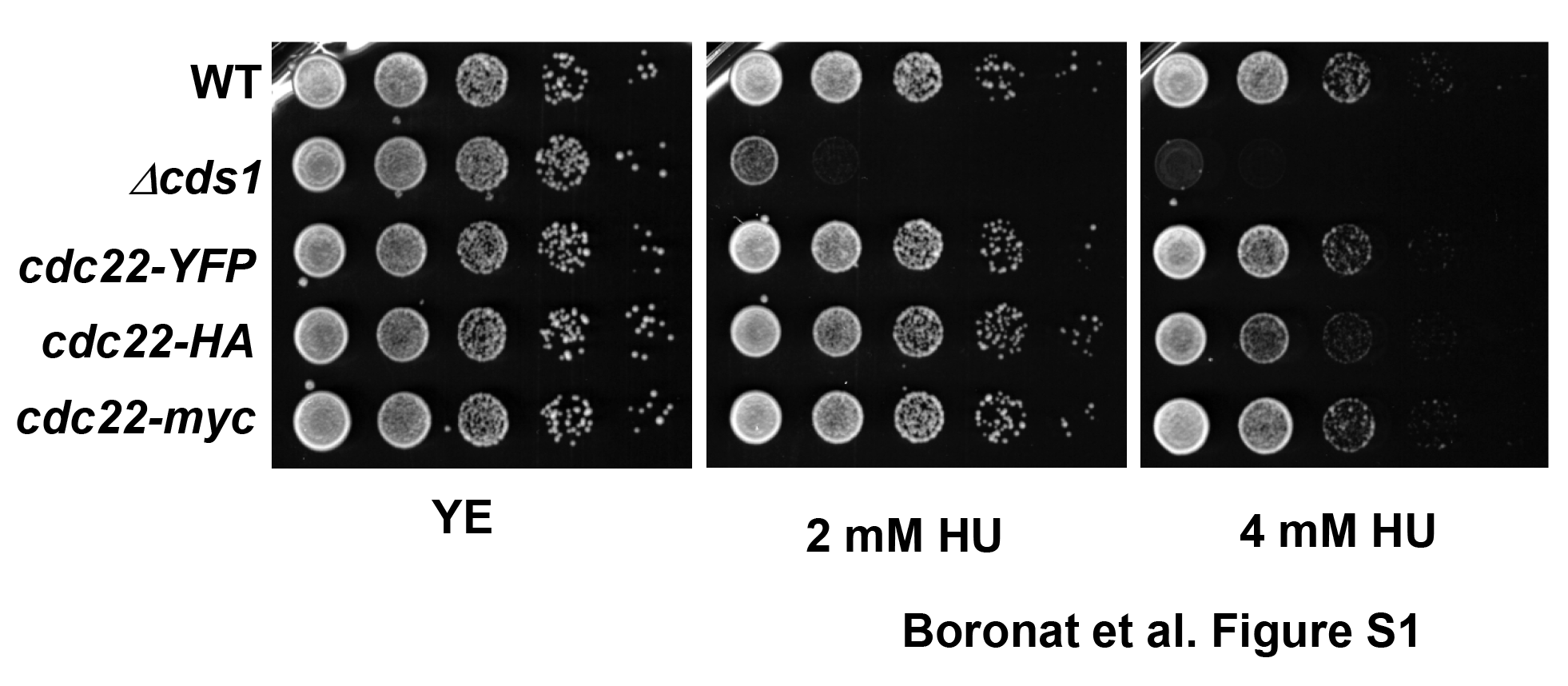

Supplement: S1 Fig — Serial dilutions of strains 972 (WT), JA1158 (Δcds1), SB62 (cdc22-YFP), SB104 (cdc22-HA) and SB212 (cdc22-myc) were spotted on agar plates without (YE) or with 2 and 4 mM HU, and grown for 3 days at 30°C. (TIF) [file pgen.1006858.s001.tif]

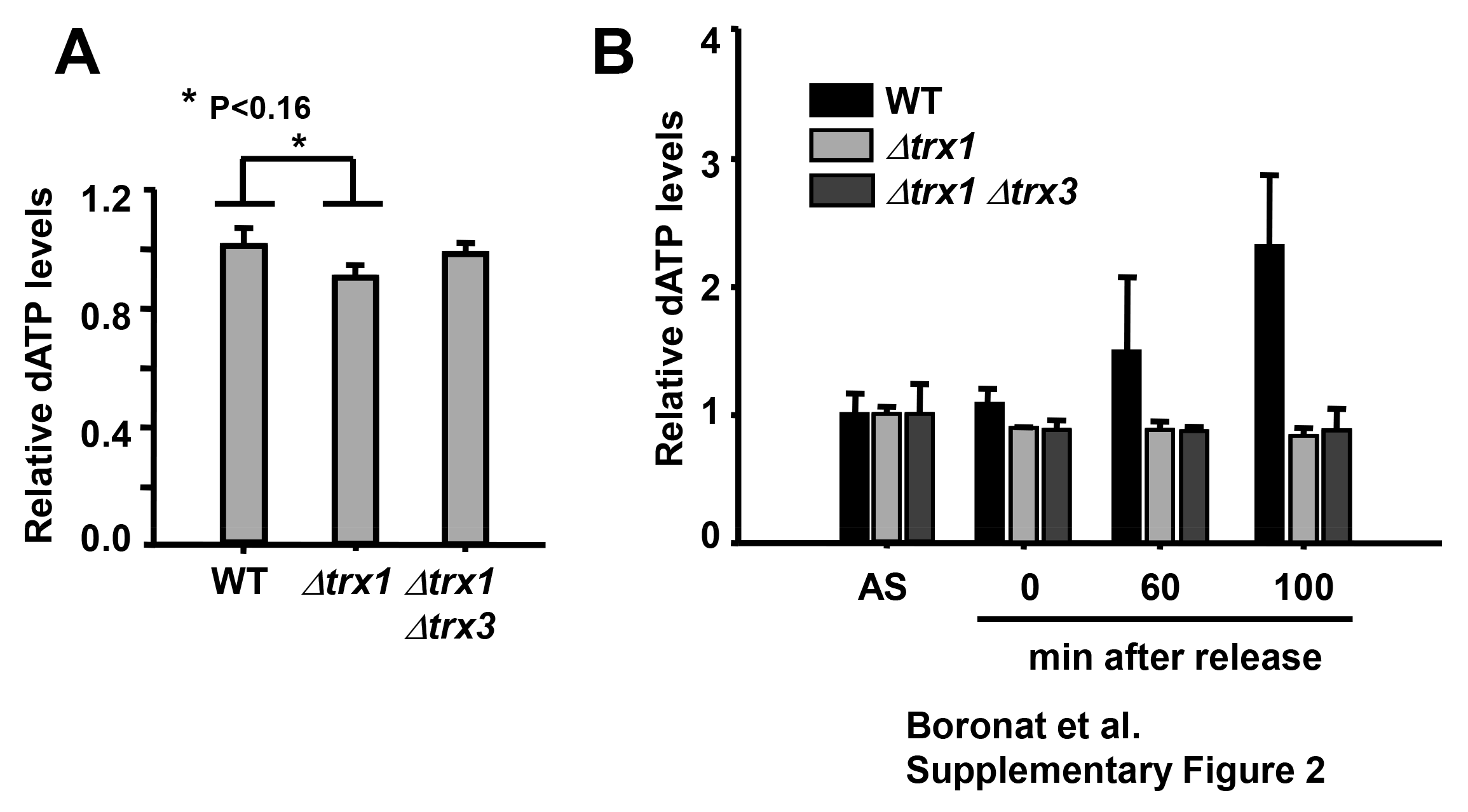

Supplement: S2 Fig — (A) dATP levels in wild-type and Trx mutants. Strains SB117 (WT), SB137 (Δtrx1) and SB138 (Δtrx1 Δtrx3) were grown in YE and dATP levels were determined by the DNA polymerase-based enzymatic assay. The values are represented relative to those of the wild-type strain. Error bars (SEM) from three independent experiments are shown. (B) dATP levels of cell cultures as in Fig 2A at the indicated times points were determined and represented as in Fig 1E. (TIF) [file pgen.1006858.s002.tif]

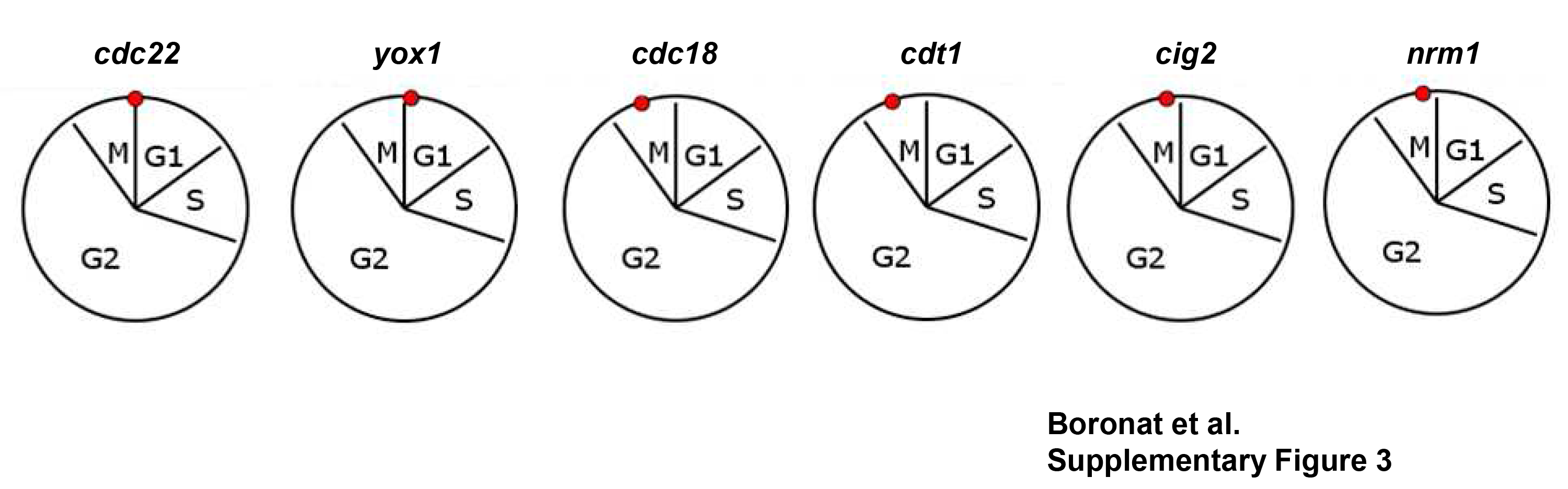

Supplement: S3 Fig — Combined peaktime of the S phase transcripts cdc22, yox1, cdc18, cdt1, cig2 and nrm1. Data obtained from the database Cyclebase 2.0. (TIF) [file pgen.1006858.s003.tif]

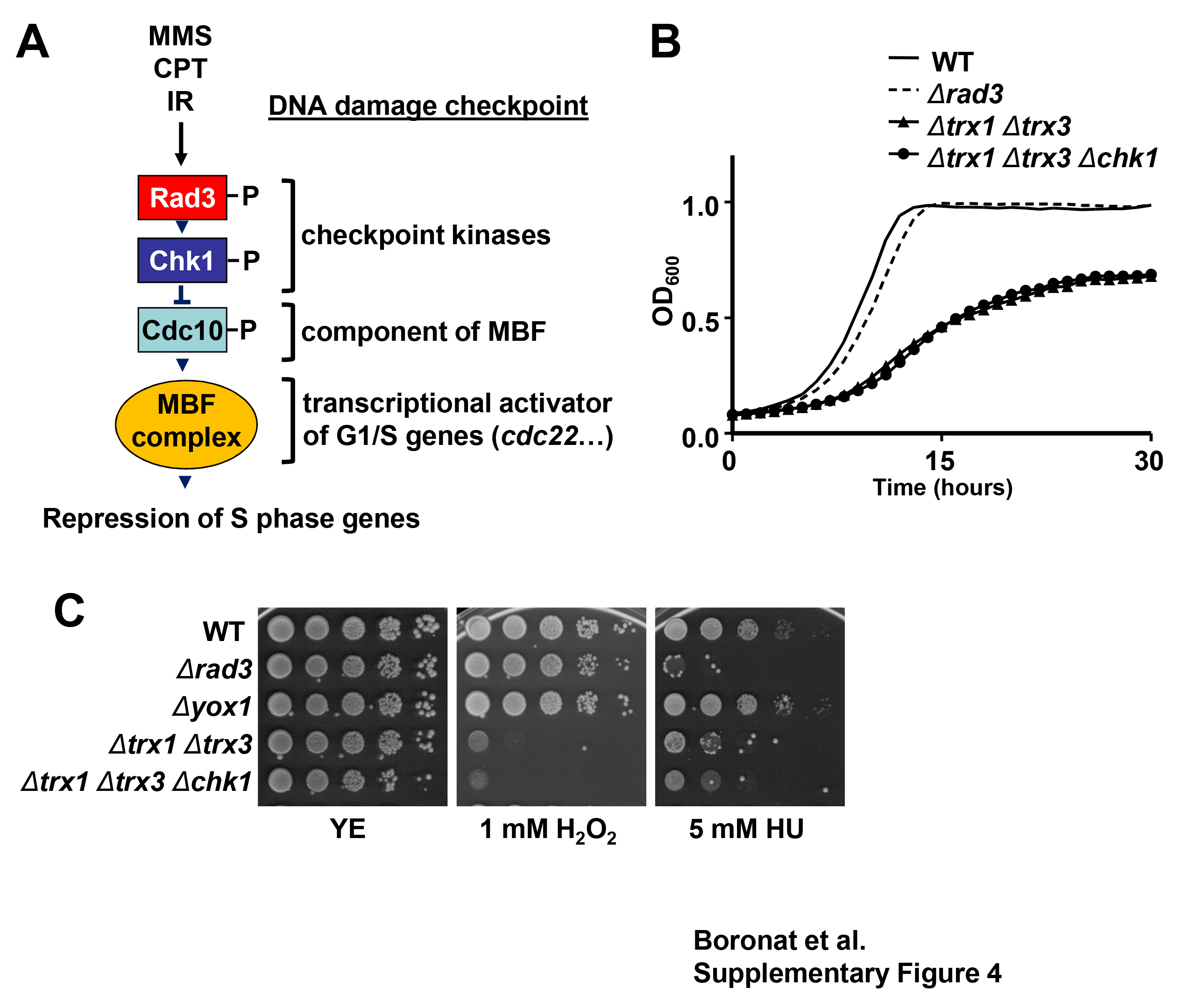

Supplement: S4 Fig — (A) Scheme depicting the activation of Chk1 upon DNA damage. DNA damage activates the kinases Rad3 and Chk1. Cdc10, a member of the MBF complex, is then phosphorylated by Chk1, resulting in its release from chromatin and leading to repression of MBF dependent genes. (B) Δchk1 does not display genetic interaction with Δtrx1 Δtrx3. Growth of 972 (WT), JA804 (Δrad3), SG248 (Δtrx1 Δtrx3) and AD151 (Δtrx1 Δtrx3 Δchk1) cells was monitored by recording the OD600 for a period of 30 hours. (C) Serial dilutions of strains 972 (WT), JA804 (Δrad3), JA795 (Δyox1), SG248 (Δtrx1 Δtrx3) and AD151 (Δtrx1 Δtrx3 Δchk1) were spotted on agar plates without (YE) or with 1 mM H2O2 or with 5 mM HU, and grown for 3 days at 30°C. (TIF) [file pgen.1006858.s004.tif]

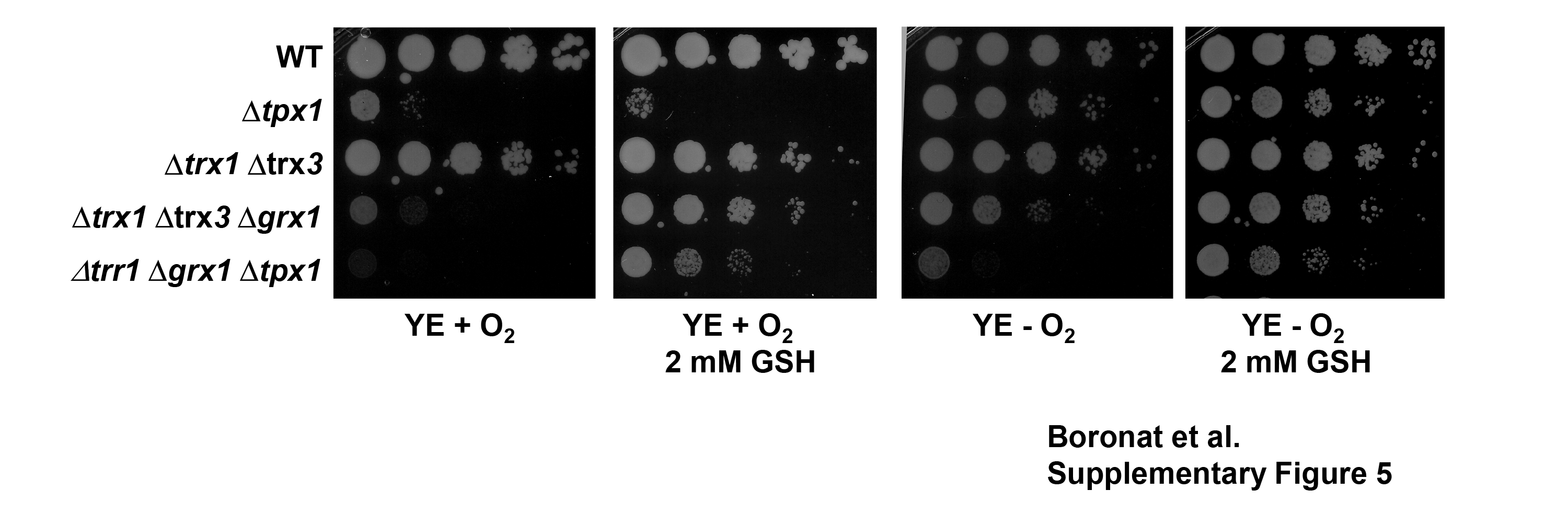

Supplement: S5 Fig — Serial dilutions of strains 972 (WT), SG4 (Δtpx1), SG248 (Δtrx1 Δtrx3), SB304 (Δtrx1 Δtrx3 Δgrx1) and MC144 (Δtrr1 Δgrx1 Δtpx1) were spotted on YE agar plates without or with 2 mM GSH under semi-anaerobic (-O2) or aerobic (+O2) conditions and grown for 3 days at 30°C. (TIF) [file pgen.1006858.s005.tif]
