# Supplementary material for: Lack of a peroxiredoxin suppresses the lethality of cells devoid of electron donors by channelling electrons to oxidized ribonucleotide reductase
Source: PLoS Genet. 2017 Jun 22;13(6):e1006858. doi: 10.1371/journal.pgen.1006858 (PMC5501661; doi:10.1371/journal.pgen.1006858)
Supplement: S1 Table — (PDF) [file pgen.1006858.s006.pdf]

Supplemental Information, Boronat et al. 2017

S1 Table. Phenotypes of strains mutated in different components of the Trx and Grx systems.

**Strains**	**Genotype**	**Viability**	**Aerobic/semi-anaerobic plates**[a]	**Fitness**[b]
MJ15	*Δtrx1*	Yes	Aerobic	***
MJ16	*Δtrx2*	Yes	Aerobic	****
MJ13	*Δtrx3*	Yes	Aerobic	****
SG248	*Δtrx1 Δtrx3*	Yes	Aerobic	**
IC38	*Δgrx1*	Yes	Aerobic	****
IC44	*Δtrx1 Δgrx1*	Yes	Aerobic	***
SB160	*Δtrx1 Δtrx3 Δgrx1*	Yes	Anaerobic	*
SB226	*Δtrx1 Δtrx2 Δtrx3 Δgrx1*	Yes	Anaerobic	*
MC122	*Δtrx1 Δtrx3 Δgrx1 Δgrx2*	Yes	Anaerobic	*
SG166	*Δtrr1*	Yes	Aerobic	**
AD131	*Δtrr1 Δgrx2*	Yes	Aerobic	**
AD84	*Δpgr1*	Yes	Anaerobic	*
	Δtrr1 Δgrx1	No		
	Δtrr1 Δgcs1	No		
MC125	*Δpgr1 Δtrx1*	Yes	Anaerobic	*

[a]Requirement for aerobiosis or anaerobiosis for cell survival on YE plates. Anaerobic strains require anaerobic conditions for growth on solid YE media.
[b]Fitness of each strain was classified in four different groups based on two criteria: growth rate in aerobic liquid cultures and survival in plates with different H_2O_2 concentrations.
**** Wild-type growth rate and wild-type tolerance to H_2O_2
*** Wild-type growth rate and mild sensitivity to H_2O_2
** Slower than wild-type growth rate and severe sensitivity to H_2O_2
* No growth under aerobic conditions. Cell cultures have to be anaerobic or supplemented with GSH. No survival on plates containing H_2O_2
