# Supplementary material for: Lack of a peroxiredoxin suppresses the lethality of cells devoid of electron donors by channelling electrons to oxidized ribonucleotide reductase
Source: PLoS Genet. 2017 Jun 22;13(6):e1006858. doi: 10.1371/journal.pgen.1006858 (PMC5501661; doi:10.1371/journal.pgen.1006858)
Supplement: S2 Table — (PDF) [file pgen.1006858.s007.pdf]

Supplemental Information, Boronat et al. 2017

S2 Table. Strains used in this study

Strain	Genotype	Origin
972	*h⁻*	[1]
SB104	*h⁺ cdc22-HA::natMX6*	This work
SB105	*h⁻ cdc22-HA::natMX6 trr1::ura4 ura4-D18*	This work
SB106	*h⁻ cdc22-HA::natMX6 trx1::ura4 ura4-D18*	This work
SB110	*h⁺ cdc22-HA::hphMX6*	This work
SB112	*h⁻ cdc22-HA::natMX6 tpx1::kanMX6*	This work
SB115	*h⁻ cdc22-HA::hphMX6 trx3::kanMX6 leu 1-32*	This work
SB121	*h⁻ cdc22-HA::hphMX6 trx1::natMX6 trx3::kanMX6*	This work
SB132	*h⁻ cdc22-HA::hphMX6 trx1::natMX6 grx1::kanMX6*	This work
SB133	*h⁻ cdc22-HA::hphMX6 trx1::natMX6 trx2::kanMX6*	This work
SB134	*h⁻ cdc22-HA::hphMX6 trx2::kanMX6*	This work
SB140	*h⁺ cdc22-HA::hphMX6 grx1::kanMX6*	This work
SB111	*h⁻ cdc22-HA:: natMX6 grx4::ura4 ade6-704 leu1-32*	This work
SB131	*h⁻ cdc22-HA:: hphMX6 grx2:: kanMX6*	This work
AD178	*h⁻ cdc22-HA:: natMX6 grx3:: kanMX6*	This work
AD181	*h⁺ cdc22-HA::hphMX6 grx5:: kanMX6*	This work
SB308	*h⁻ cdc22-HA::natMX6 trx1::ura4 trx3::hphMX6 grx1::kanMX6 ade6-704 leu1-32 ura4-D18*	This work
AD104	*h⁺ cdc22-HA::hphMX6 pgr1::natMX6*	This work
SB198	*h⁻ cdc22-HA::natMX6 yox1-13myc::kanMX6 cds1::hphMX6*	This work
SB117	*h⁻ cdc25-22 cdc22-HA::natMX6*	This work
SB142	*h⁻ cdc25-22 cdc22-HA::hphMX6 trx3::kanMX6*	This work
SB137	*h⁺ cdc25-22 cdc22-HA::natMX6 trx1::ura4 ura4-D18*	This work
SB141	*h⁻ cdc25-22 cdc22-HA::hphMX6 trx1::natMX6 grx1::kanMX6*	This work
SB138	*h⁻ cdc25-22 cdc22-HA::hphMX6 trx1::natMX6 trx3::kanMX6*	This work
SB149	*h⁻ cdc25-22 cdc22-HA::natMX6 yox1-13myc::kanMX6*	This work
SB171	*h⁺ cdc25-22 cdc22-HA::natMX6 cds1::kanMX6*	This work
SB199	*h⁻ cdc25-22 cdc22-HA::natMX6 trx1::ura4 yox1-13myc::kanMX6 cds1::phpMX6 ura4-D18*	This work
SB200	*h⁻ cdc25-22 cdc22-HA::natMX6 yox1-13myc::kanMX6 cds1::hphMX6*	This work
SB150	*h⁻ cdc25-22 cdc22-HA::natMX6 trx1::ura4 yox1-13myc::kanMX6 ura4-D18*	This work
SB153	*h⁻ cdc25-22 cdc22-HA::natMX6 trx1::ura4 trx3::hphMX6 yox1-13myc::kanMX6 ura4-D18*	This work
SB62	*h⁻ cdc22-YFP::kanMX6*	This work
SB212	*h⁺ cdc22-myc::natMX6* ade6-M210 *ura4-D18 leu1-32*	This work
AD151	*h⁻ trx1::ura4 trx3::hphMX6 chk1::natMX6 ura4-D18*	This work
JA795	*h⁻ yox1::kanMX6*	This work
JA804	*h⁻ rad3::kanMX6*	This work
JA1158	*h⁺ cds1::natMX6*	This work
SG4	*h⁺ tpx1::natMX6*	This work
SG70	*h⁻ trx1::ura4 ura4-D18*	This work
SG166	*h⁺ trr1::natMX6*	This work
SG169	*h⁺ trr1::kanMX6*	This work
SG195	*h⁻ grx1::kanMX6 trr1::natMX6 tpx1*^{STOP}	This work
SG248	*h⁻ trx1::ura4 trx3::hphMX6 ura4-D18*	This work
MC122	*h⁻ trx1::kanMX6 trx3:: natMX6 grx1::hphMX6 grx2:: kanMX6*	This work
MC125	*h⁻ trx1:: kanMX6 pgr1:: natMX6*	This work
MC138	*h⁻ tpx1::natMX6 grx1::hphMX6*	This work
MC144	*h⁻ trr1::kanMX6 tpx1::natMX6 grx1::hphMX6*	This work

MJ13	*h^+ trx3/txl1::kanMX6*	This work
MJ15	*h^+ trx1::kanMX6*	[2]
MJ16	*h^- trx2::kanMX6*	[3]
AV18	*h^- sty1::kanMX6*	[4]
IC38	*h^- grx1::kanMX6*	[3]
IC44	*h^- trx1::natMX6 grx1::kanMX6*	[3]
IC76	*h^+ trx3::kanMX6 trx1::natMX6 ura4-D18 leu1-32*	[2]
IC146	*h^- trx1::natMX6 trx2::kanMX6 trx3::hphMX6*	This work
SB34	*h^- grx1::hphMX6*	This work
SB160	*h^- trx3::kanMX6 trx1::natMX6 grx1::hphMX6*	This work
SB304	*h^- trx3::hphMX6 trx1::ura4 grx1::kanMX6 ura4-D18*	This work
SB226	*h^- trx1::natMX6 trx2::kanMX6 trx3::kanMX6 grx1::hphMX6*	This work
AD84	*h^- pgr1::natMX6*	This work
AD131	*h^+ trr1::natMX6 grx2::kanMX6*	This work
AD172	*h^+ trx1::ura4 trx3::hphMX6 grx1::kanMX6 tpx1::natMX6 cdc22-HA::natMX6 ura4-D18*	This work
AD175	*h^- trx1::ura4 tpx1::natMX6 cdc22-HA::natMX6 ura4-D18*	This work
AD176	*h^- trx1::ura4 trx3::hphMX6 tpx1::natMX6 cdc22-HA::natMX6 ura4-D18*	This work

SUPPLEMENTAL REFERENCES

1. Leupold U (1970) Genetical methods for *Schizosaccharomyces pombe*. Methods Cell Physiol 4: 169-177.
2. Garcia-Santamarina S, Boronat S, Calvo IA, Rodriguez-Gabriel M, Ayte J, et al. (2013) Is oxidized thioredoxin a major trigger for cysteine oxidation? Clues from a redox proteomics approach. Antioxid Redox Signal 18: 1549-1556.
3. Calvo IA, Boronat S, Domenech A, Garcia-Santamarina S, Ayte J, et al. (2013) Dissection of a redox relay: H2O2-dependent activation of the transcription factor Pap1 through the peroxidatic Tpx1-thioredoxin cycle. Cell Rep 5: 1413-1424.
4. Zuin A, Vivancos AP, Sanso M, Takatsume Y, Ayte J, et al. (2005) The glycolytic metabolite methylglyoxal activates Pap1 and Sty1 stress responses in Schizosaccharomyces pombe. J Biol Chem 280: 36708-36713.
